# Extra Supplement—The Nursing Mirror

**Published:** 1889-01-12

**Authors:** 


*The Hospital*,
JANUARY 12, 1889.

[*Extra Supplement.*

### The Nursing Mirror.

All Contributions for this Supplement should be addressed "The Nursing Mirror," care of The Editor, 38, Old Jewry, E.C.

### En Passant.

SHORT ITEMS.—A delightful entertainment was given to the nurses at Great Ormond Street on January 3rd. —The poor nurses at Birmingham have to polish the ward floors as a commencement of their day's labour. In Parisian hospitals men polish the floors.—An extra nurse has been appointed at Inverness Infirmary, to meet the requirements of the enlarged building.—A new nursing institute has just been opened at Sunderland.

KINDLY GREETING.—The friendly relations of many of our readers towards ourselves has been specially shown this Christmas, and THE HOSPITAL has come in for quite a large share of seasonable good wishes and congratulations. It is very pleasant to us to learn that our readers appreciated our efforts to aid and interest them, and we thank them heartily for their kind thoughts. One very pretty card which came to the office bore two lines from Tennyson we found specially cheering :—

"Blest be Heaven that brought thee here,
To warm my cold heart with a friend."

RELIGIOUS, OR LAY NURSES?—The lay nursing of French hospitals does not seem to gain ground in public favour. The *Times* correspondent says : "The laicization of the hospitals has provoked, and still provokes, extreme irritation. I have seen hundreds of patients, doctors, and visitors who have had the means of comparing the services of the sisters and the lay attendants, and all describe the laicization as a calamity for the sick. 'I left cured,' said a patient to me, 'but exasperated.' The grumblings of these patients have an immense influence, for men of the working class occupy the hospital beds and communicate their anger to the mass of the voters. The stupid anti-religious campaign undertaken by persons whose only idea of political science was to persecute and to place citizens outside the law has been one of the great causes of dissatisfaction."

GLASGOW ROYAL INFIRMARY.—A pleasant custom exists at this infirmary of holding a meeting of managers and nurses on New Year's Day. On January 1st last Sir James King, Lord Provost, presided at this annual meeting, and wished all present a happy New Year. The speech which chiefly interested the nurses was that of Professor James Dunlop, whose surgical skill appeals much of the womanly instincts of many who wait on him. Professor Dunlop mentioned that 22 nurses trained at the infirmary now held the posts of matrons elsewhere, and he urged on the committee that those nurses who showed natural aptitude should be trained in hospital economics and management specially to fill these posts of trust. Professor Dunlop then proceeded to urge on his hearers the importance and advantage of joining the National Pension Fund for Nurses. There seems to be an absurd idea amongst a few of our Scotch sisters, that for some reason they are not likely to secure so much benefit from the fund as the English nurses will. This is quite an erroneous impression, and we hope that after Professor Dunlop's kind and hearty advice, all fears of this sort will be removed. The Fund is truly "National," and as it is in some sort a bond of fellow interest amongst all its members, we long for more of our friends over the border to join our lists. It is such a pity there is a talk of founding a small pension fund for Liverpool nurses ; co-operation in the National Fund would be so much better for all concerned.

TO THE MISSION FIELD.—Stratford-on-Avon this month loses one of its most beloved inhabitants. On January 12th, Miss Smith, the matron of the hospital, sails for Zanzibar to enter on missionary work under Bishop Smythies. Great though the loss will be to Stratford, its people are proud that the spirit of self-sacrafice has thus claimed one from their midst, and all their good wishes and prayers will follow Miss Smith in her labours in Africa. The same high principle and clear sense of duty which has made all Miss Smith's nursing work so fruitful of good over here, is sure to sustain and help her in her labours in the mission field ; we can scarcely grudge such a noble character to work which will call forth all its unusual and splendid powers. Practical tokens of esteem have not been denied to Miss Smith ; a cheque and a surgical case from nurses and friends, and a travelling clock from the medical staff have been presented to her.

PROBATIONERS A BLESSING.—The poor pros. who are at present in such a mournful frame of mind may be cheered to hear that their services are appreciated at Boston Cottage Hospital. Some time ago this hospital was nursed chiefly by paid nurses, but lately, under Miss Heanley's management, a new system has been tried, and a succession of young ladies have been trained as probationers, who not only give their services, but pay for their maintenance also. In this way a better class of workers is obtained, much to the satisfaction of the patients. On the retirement of one of the probationers, who had been trained as a nurse in the hospital, to another appointment, twenty-five of the old patients attended at the hospital as a deputation to express their gratitude for the kindness and skill shown to them, and presented her with a waist-belt with silver mountings, provided with the usual surgical instruments worn by nurses. One or two of the deputation had lost their legs, two had each only one arm, and all had recovered, while in the hospital, from serious and dangerous illnesses. The saving to the hospital of this system of nursing cannot be over-estimated, as the late enlargements and improvements have left the hospital in debt, and funds are urgently needed.

NOVEL NURSES.—John Strange Winter has certainly the knack of telling short stories, and she does not scorn to avail herself of the pathetic touches which hospitals can afford her. In the shilling volume *On March* there was an army sister called Sister Myra, to whom the Netley nurses most stongly objected. In spite of any amount of sentiment thrown into the character, they rudely refused to recognize this Sister Myra, and John Strange Winter had to append a note to the book, in which she disclaimed having tried to draw a type of a class, and admitted she had but her heroine in the gray garb merely to meet the exigencies of the situation. Why shouldn't a pretty little immoral gardener's daughter be turned into an army sister to suit the conveniences of a novelist? We confess, though we are not surprised that the Netley nurses were highly indignant, and inclined to quarrel with the licence allowed to fiction. In a later volume "Poor Dick," the authoress has been wiser, and has apparently taken the trouble to visit Charing Cross Hospital before placing her hero and heroine within its walls. There is even an illustration of a sister wearing a cap not unlike those actually used, which is a great concession towards reality. The nurse doesn't speak very good grammar, but still we suppose we must be grateful to John Strange Winter for allowing her to be respectable, and remember that after all she is only a novel nurse, not a real one.

### Original Articles.

### LADY GUARDIANS.

Perhaps some of our readers may wonder what on earth Lady Guardians have to do with nursing; but if so, it will only be those who have never worked in a workhouse infirmary. The number of sick in our poor-law infirmaries is something enormous, and many of them find shelter there through no fault of their own. For able-bodied paupers we have no sympathy and no interest, but for the poorest of our sick we have much compassion. The weakly ones of this world for whom the struggle for existence is too sharp and severe, and whose weary days of poverty and pain draw to an end in a workhouse infirmary, should surely be granted as easy a death as possible. Life has dealt hardly with them; she has no room for these children of her's on whom disease has fastened; they must fight while they can, and fall when the last remnant of strength has passed. Let us place them gently in their beds then, let us ease the pain and soothe the wrinkles from the weary brow, let there be a little pause of peace in which to contemplate eternity, before the spirit departs to God who gave it.

Of course those who have nursed in workhouse infirmaries will say there are many of their charges who, in spite of bodily ailments, yet call forth no pity. Yet it is so difficult for us to realise the temptations of hunger and want, that we dare not judge these our brethren. Perhaps if our lives had been as void of all beauty as theirs, our words and actions would also have been coarse and rude. Such as they are, these infirmary patients, they are children of God; the very lowest and vilest is our sister; and if in their hurried striving lives there has been no time for them to think, no time for them to dream of good, nothing but a fight for bread; all the more reason that ere the end come they should be lovingly tended, that they may comprehend in some slight measure the great future before them, and choose the better part.

And here it is that the Lady Guardian can step in and speak for her poor sisters. Through many years of men guardians things have gone a weary course: plenty of care devoted to such subjects as they understood and cared for, but complete ignoring being meted to the subjects which specially need a woman's tact and skill. With our workhouse population always largely feminine, and our infirmaries always requiring some sort of nursing, it is only just that on every board there should be at least one woman. Men guardians are worse than the Egyptians; they require scrubbing done without brushes; they want poultices made without linseed; they cannot see the necessity for pails, and pots, and pans. Their minds soar above these trivialities, and the nurse asked to nurse without means or appliances, scarcely knows whether to laugh or to cry. Some of the stories these nurses tell are truly ludicrous, if our only object were to make our readers laugh; but we wish to rouse their sympathy as well. One infirmary was always full of ophthalmia, and the nurse struggled against the numerous cases in vain; at last, in answer to questions to the children, she discovered that a single towel was used every morning by forty children, many of whom had ophthalmia. Another nurse discovered that not a single pocket handkerchief was supplied to the inmates of a certain workhouse, and that her efforts for cleanliness in the wards were impossible under the circumstances. Again, the state of the bedding used often to be far too lively for description, men guardians having walked down the dormitories without ever dreaming of inspecting it. Children were often unhealthily and indecently clothed, and supplied with no nightgear, so that they were positively trained in reprehensible practices. The frequent feeding on boiled meat and no vegetables or puddings is another grievance Lady Guardians can remedy, especially with regard for the cooking of food for the infirmary, which is constantly lacking in nourishment and variety. Then there are the lying-in wards, which ought to be made the subject of much attention by the Lady Guardian, who should see that a proper nurse is provided, and that she is properly treated. There are hundreds of points about workhouse management which are utterly unsuited to be brought before a body of men, and which need a woman's special knowledge to be dealt with properly. How can you expect a male guardian to inspect the tubes of the babies' feeding bottles, and see that they are not foul?

Granted, then, that Lady Guardians are a blessing all round, more especially are they so where some brave woman has undertaken the post of nurse to a workhouse infirmary. Probably she will be more poorly paid than in a more comfortable situation, but the greater power of doing good has led her to put her hand to this special work. The influence of Miss Agnes Jones is still felt by many, who, knowing what she underwent, and how glorious was her victory, long to fight on the same battle-field; and follow, even though far off, in her footsteps. A nurse in this position is almost isolated: she is like the settler in the Colonies, who has to fight single-handed against Nature, and clear every inch of ground of a wealth of weeds and briars. Used to all the appliances of a hospital, to the companionship of other nurses, to the constant attendance of doctors who take all responsibility, she suddenly finds herself in a strange, uncivilized land as it were, where she has to make bricks without straw. Day and night all work and all responsibility is on her shoulders, and she has no one to turn to for help or advice. She looks in vain for lint or bandages, in vain for carbolic; her food she has to cook herself in the ward, and of course has then no appetite to eat it. Her patients need beef tea, clean sheets, brushes and combs, but she cannot get them for them, and, as before stated, her interviews with the Board leave her doubtful whether to laugh or cry.

In such a fix if a Lady Guardian is elected the nurse is sure of immediate sympathy and future help. Here is someone to whom she can explain her difficulties, and who can appreciate her wants. No longer will tea be made by stewing six ounces of tea in six gallons of water for two hours; no woman on earth would countenance this ruining of her favourite beverage; and all the rest of the cooking, especially for the infirmary, will be overhauled. The subject of nursing will come to the fore; the sleepy old doctor who has grown used to these things will be roused to believe that there is no reason why paupers should die without proper attention, and our brave nurse will probably be able to clear her ground and sow her seed, and in time raise a crop of blessings as a reward for her labours.

And in those districts where there is no trained nurse the Lady Guardian will soon discover the want, and do her best to oust the Sairey Gamp. It is one of the first points a Lady Guardian should attend to; the nursing in the workhouse infirmary, and it the one that most needs rectifying both in London and the provinces. A great change for the better has been inaugurated by the Association for Promoting Trained Nurses in Workhouse Infirmaries, of which Miss Wilson is secretary; but this society wants the support of the Society for Promoting the Return of Women as Poor Law Guardians. Without ladies on the Board, the trained nurse is often unable to do much good. We all of us have a certain moiety of influence, and if any of us have hitherto never exercised it towards the return of Lady Guardians, believing that to be a question without our province, let us remedy our mistake in future; remembering that, without Lady Guardians, it is almost impossible to secure proper nursing for our paupers when their last and bitterest ill has befallen them, and their pain and poverty are culminating in the climax of death.

### National Pension Fund for Nurses.

An important decision has been made and acted upon by Mr. James E. Mathieson, the Treasurer of the Mildmay Nursing Home, Mildmay Park, N., which employs upwards of one hundred nurses. Some years ago a scheme for providing the nurses with pensions after fifteen years' service, and subject to other conditions, was instituted. With a view to placing these arrangements on a sounder and more satisfactory basis, negotiations were entered into with the Council of the National Pension Fund, which have now been happily concluded. Mr. Mathieson has paid five hundred pounds (£500) into the National Pension Fund, and, in addition, has agreed to pay £150 per annum for a term of years, for those of the Mildmay nurses who are embraced by the old scheme, with £2 per nurse per annum for all the others who have or may join since the old offer was withdrawn. Mr Mathieson is most anxious to encourage thrift amongst the Mildmay nurses, and every one for whose benefit he pays £2 a-year is expected to pay at least an equal amount into the National Fund out of her earnings as a nurse. Several other hospitals and institutions are now considering how they can best avail themselves of the National Pension Fund for Nurses in the interest of the staff they employ. The only serious criticism of the Fund ever made amounted in effect to a statement that the rates in the tables were unnecessarily high. It is now realised by most institutions and nurses that, as the Fund is entirely mutual, there is no real force or practical value in such criticisms, seeing that the annuitants will receive back every penny that their money will purchase, and large bonuses from the free gifts of the public in addition. The Fund has undertaken a new class of business, the risks of which could not be precisely ascertained at the outset, so the actuary was bound to make the Fund absolutely safe, otherwise Lord Rothschild, Mr. Junius S. Morgan, Mr. H. Hucks Gibbs, Mr. E. Hambro, Mr. W. H. Burns, Mr. Alfred de Rothschild, and other eminent financiers could not have identified themselves with the Fund, as they have done from the outset. A guarantee of more than is promised, absolute safety with good interest for all their savings, and the prospect of substantial bonuses in addition, have overborne the mild criticisms of a few ill-disposed persons; and so nurses are joining the Fund in increasing numbers every day. Twenty-five new policies were taken out in a single day last week; and the managers of the institutions concerned are manifesting a desire to ascertain to what extent they can assist their employés to procure pensions through the National Pension Fund for Nurses.

### THE BRADFORD INFIRMARY.

The nurses of the Bradford Infirmary were entertained at a Christmas party, given by the lady superintendent and the resident surgical staff, on Thursday evening, January 3rd. The evening passed very pleasantly, with music, vocal and instrumental, games, and round dances. Supper was served in the board-room at 9.30, after which the evening's enjoyment ended with Sir Roger and the National Anthem. The warmest thanks of the nurses are due to the lady superintendent, the house surgeons, and other friends, who so kindly contributed to the evening's entertainment.

### A Christmas Holiday.

### Part I.

Nurse Sadler and I had an invitation to spend Christmas at Brighton, so we applied to the superintendent of the institution for which we both work, and got leave of absence from Saturday, December 22nd, to Thursday, the 27th. We were going to some old friends of ours, who were rather dull but worthy people, and we both came to the conclusion that six days of their company was more than was necessary, and therefore we talked over the wild scheme of walking to Brighton. Neither of us had had tiring cases lately, but we had both been attending wearisome chronic cases, which confined us much to the house. "I do want fresh air so badly," said Nurse Sadler, "and old Mrs. Lloyd will only drive us about in a brougham when we get down to Brighton. I feel quite fit for a three days' tramp if you do."

We had a long discussion about the route; I had friends at Guildford who would put us up for the night, so we finally decided to march there the first day, and then go on by Horsham. I bargained for starting beyond the region of villadom, so on Saturday morning we took train to Richmond to walk across the park.

"Now we are here," suggested Nurse Sadler as we got out at Richmond Station, "don't you think we ought to see th hospital?"

"Where is the hospital?" I asked a porter.

"Only five minutes' walk, mum; straight down that little road on your right."

So we walked away down a clean little back street till we came to a pleasant-looking red brick building facing the old Deer Park. We walked boldly into the hall and asked for the matron, but Mrs. Liddall was too occupied to see us, so we asked for a nurse who had trained with us some years before, and she came down and showed us over the building. It was a neat little hospital with a large ward of about 20 beds for men, and a similar one for women—no children's ward, as no one under six is admitted. The little theatre had a single ward off it, into which a patient could be wheeled, and left undisturbed for days. Behind the hospital was a nice bit of garden, which the nurses enjoyed. A house surgeon lives at the hospital.

"We want a nursing institution in Richmond," said our guide; "there are several trained nurses living here, and well occupied, but sometimes a doctor will spend hours in seeking one, and finally have to send to London."

We did not waste much of our time at the hospital, but hurried off up to the park, where the soft breezes welcomed us. It was not in the least like Christmas weather; the wind was warm and mild, and the sun shone softly on the rich green grass. We trudged along manfully all the morning, and thoroughly enjoyed the exquisite country we found after passing Claremont. It was a regular pine country, and the red stems of the great fir trees, and the silver bark of the graceful birches met us at every turn. There were no hedges at the road-side, and every now and then we wandered into the woods, or across the picturesque bits of sandy heath, and found treasures in the shape of huge red toadstools, or a few pale primroses. In one part we came across a lake quite surrounded with pine woods, and the scenery was so Scotch we could scarcely realise we were in Surrey. About two o'clock two very weary and hungry travellers arrived at Ripley, after five hours hard walking. We found a quaint old inn, and were shown into the parlour behind the bar, where a substantial lunch was placed befors us. Two dogs and a cat shared the room with us, and just outside the low, open window, gathered a crowd of hens, turkeys, pigeons, two jackdaws, and a disreputable-looking magpie. We enjoyed both our lunch and our company, and then started on our way towards Guildford once more. The country now was not so beautiful; we had left the sandy soil and the roads were muddy and common-place.

*(To be continued.)*

### Everybody's Opinion.

### HOSPITAL SWEATING.

A Matron writes : I have been reading your article "Philanthropic Sweating." I had not seen the letter in the *Daily News*, but I think the remarks which you quote are most unnurselike, and must come from someone who is very discontented. But whilst there is, no doubt, a very great improvement in many of our hospitals, I do believe in others matters could not be much worse than what they are. Last summer I many times made my dinner from potatoes and butter because the meat was simply rotten. I once did so on three consecutive days ; in fact, we used to hail with delight the appearance of meat which we could eat with a relish. Green vegetables we seldom saw, and we got a small tart once a week. There was no nurses' dining-room ; we had our meals in our own sitting-room in the block. The hospital comprised four blocks. The others cooked their own food, a most objectionable plan, I consider, for either your patients or your dinner must meet with neglect. My dinner was consequently cut off, some potatoes dabbed on the top of the meat, placed between half-cold plates, and carried across the grounds from the administration block. In that same hospital margarine was supplied in place of butter to the patients and servants. In county asylums, the food, as a rule, is abominable. The best I have known was at the Berks County at Moulsford, near Wallingford. There was plenty of variety, of good quality, and well served. The nurses' accommodation, too, was excellent ; but, then, I did not consider that other things were equal. It was when Dr. G—— was Medical Superintendent, and he was most difficult to please ; but I suppose it was owing to the disease, which was easy of detection even then, with which he ultimately died at a private asylum at Sevenoaks. I have spoken of bad food at one hospital. I think I ought to say that during six weeks at Winchester Hospital I found the food very good. I was only there six weeks, as I went as a private nurse to take some special cases. You say a nurses' life is hard ; I have yet to find that out. I think my lot must have fallen in pleasant places. Of course, I admit the food question ; but our lives cannot be made up of eating and drinking, and how many hundreds of people are there who would be thankful to get that which to us is coarse and unsavoury ? I think it is well to content oneself with the belief that nothing in this world can last for ever.

### MEDICAL AND SURGICAL HOMES.—FIFTEEN YEARS' EXPERIENCE.

Mrs. Alexander writes from 3, Manchester Street, Manchester Square, W.: Having seen a request in The Hospital that all reports, &c., respecting homes or institutions, should be sent to you, I write to say that I am retiring from my work in this house. It is fourteen years since I started it, at which time I believe there were only three other of these homes in this neighbourhood. Within six months two gave up, so that, with one exception, I think I may consider mine the oldest established. During these fourteen years I have received 1,054 patients, their stay varying from a few hours to 53 weeks. I might have had a greater number, but I have only six available bedrooms, each patient being in a separate room. The number of deaths have been 40, which I am afraid you will think a high percentage, but when you consider that so many suffered from internal complaints and underwent abdominal section in some form, it is hardly to be wondered at. I think these are all the particulars I have to give, but should you wish for further information, I shall be happy to give it. I feel great sorrow at giving up a profession which has so much that is pleasant in it, though at the same time it is full of continual anxiety. But there is great satisfaction in looking back upon the fourteen years, and knowing that the success has been so great that, as I have already said, I am enabled to retire into private life. I have no objection that you should publish the facts as I have stated—in fact, I would rather you did, as I may soon be inserting an advertisement in The Hospital respecting some medical and surgical appliances I wish to sell; and I should be sorry for that to appear first, as it might give your readers a wrong impression about me.

### REST AND EXERCISE.

M. B. writes: In reading your article on "Rest and Exercise," I can affirm it is only too true what the nurse writes you. In confidence I could give you the history of many of the commercial nursing institutions all over the country, worked up patiently, and perfected for their own personal benefit, and no thought for the poor nurse, who is badly paid, and harassed with many restrictions. I am a trained nursing sister, and necessity has caused me to work in several of them. My experience is that one and all are brutal—robbers of the widow and orphans. It requires a high hand to deal with them. I could give you details that would disgust you. The same hand that swept the Italian taskmaster and the German tyrant over the poor "buy a broom" girls of the past ought to attack them. When the nurses are ill in health they are sent away; when they are over thirty-five years their services are required no longer. I have said more than I had intended, but I know so many poor nurses and nursing sisters so poor and unhappy that many have talked of suicide, not being able to find employment on account of the institutions monopolising all the private nursings.

### A Nurse-Matron in Fiji!

Can any matron or lady superintendent recommend a well-trained businesslike sister or nurse of from thirty to forty for this post ? There is good work to be done, and the officials are English gentlemen, from whom a lady might expect all support and help. Salary £75, with board and residence in the hospital, which has accommodation for seventy-eight patients, of whom seventy are natives, but there are usually not more than thirty beds occupied. Term of agreement three years, but if this is broken the passage money out must be refunded. Apply to Mr. Henry C. Burdett, The Lodge, Porchester Square, W.

### Appointments.

Coldstream Cottage Hospital.—Mrs. Humphries, a trained nurse of experience, has been appointed matron of this lately-finished hospital.

Royal United Hospital, Bath.—Miss Hodgkin has been appointed lady superintendent of nurses at this institution, out of a large number of candidates.

### Vacancies.

The following vacancies are announced :—

**Matrons.**

GatesheadChildren's Hospital ; £35.
Bootle Borough Hospital ; £50.

**Superintendent of Nurses.**

Northampton General Infirmary ; £65.

**Nurses.**

Nursing Institution, 96, 97, 98, Wimpole Street, London, W., Mr. Wilson has several vacancies for thoroughly-trained Nurses.

Belgrave Hospital for Children; one.

Dewsbury General Infirmary ; one ; £24,

Royal Albert Hospital, Devonport ; several ; £25.

Southport Nurses' Home ; several.

Royal Hospital for Women and Children.

Eltham Cottage Hospital.

Blackheath Institute ; several ; £20.

Royal Surrey County Hospital, Guildford ; £16.

Newark Hospital, £25 ; several.

Sheffield Infirmary ; £20.

Jersey Institution for Hospital-trained Nurses, £25 ; several.

St. Bede's, Swiss Cottage.

Norfolk and Norwich Nurses Home ; several.

Borough Hospital, Sheffield; several; £25 to £30.

Hatfield Broad Oak Cottage Hospital ; one ; £30.

Royal Cornwall Infirmary, Truro ; one ; £25.

Swansea Hospital ; one ; £22.

Ryde Infirmary ; £20.

Nurses' Home, Ellison Place, Newcastle-on-Tyne ; several.

Alcester Union Infirmary ; £25.

Christ's Hospital, Hertford ; £20.

Victoria Home, Bournemouth ; several.

Nottingham Nursing Institution.

Worcester Nurses Institution ; several.

Sheffield Union ; £20.

Nursing Home, Rokeby Road, Brockley, S.E. ; several.

General Nursing Institute, Henrietta Street, Covent Carden ; several.

Brdignorth Union ; £25.

**District Nurses.**

Parish of Wrexham ; £60.

Cotswold House, Gloucester ; £25.

Madeley, Staffordshire.

**Probationers.**

Lancaster Infirmary ; £13.

Royal Hospital for Women and Children, Waterloo Bridge Road.

Nurses' Home, Ellison Place. Newcastle-on-Tyne ; several.

### Notes and Queries.

**Queries.**

(97) *Home Wanted.*—Can anyone tell me of a home where a young lady recovering from hysterical paralysis could be received on moderate terms for a few months? She has been homœopathically treated, and that system would still be preferred. She requires a certain amount of attention, as she cannot dress herself or walk steadily alone.—*Nurse J.*

(98) *Author Wanted.*—Please someone tell me who wrote the line—"Strive not thyself, but other men to bless."—*Sister Kate.*

(99) *Candidates for Probationer Nursing.*—Will you kindly give me any information as to the admission, payment, and age of a nurse probationer, and the most likely hospitals to apply to ?—*Brighton.*

**Answer.**

(93) *Packing a Mental Patient.*—To pack a mental patient you require two sheets wrung out of water, whatever temperature is ordered ; place the arms straight down by the sides, and wrap the sheets securely round and round the patient, who should be placed on a bed and well covered with blankets, care being taken that the feet are kept warm ; the patient must be watched and nourishment administered frequently. Plenty of assistance should be at hand in giving a pack.

### NOTICES TO CORRESPONDENTS.

*Combination of Private Nurses for their own Protection.*—"A Private Nurse" is requested to send her name and address, not for publication, but as a guarantee of good faith. "M. Butler" is also requested to send her address.

